# Evisceration of intestines with diaphragmatic laceration and gastric perforation secondary to trauma by rhinoceros attack and its management: a case report

**DOI:** 10.1097/MS9.0000000000000214

**Published:** 2023-02-07

**Authors:** Sabin Karki, Melina Shrestha, Sunil Basukala, Suman Maharjan, Sabin Banmala, Anil Hona, Narayan Thapa, Bikash B. Thapa

**Affiliations:** a Department of Surgery, Shree Birendra Hospital, Chhauni; b College of Medicine, Nepalese Army Institute of Health Sciences (NAIHS), Sanobharyang, Kathmandu, Nepal

**Keywords:** abdominal evisceration, animal attack, rhinoceros, trauma

## Abstract

Injuries from animal attack are one of the major public health problems at present scenario globally. Proper documentation is required for the study of different types of injuries caused by animal attacks so that early intervention can be done during life-threatening conditions.

**Case Presentation:**

A 36-year-old male presented with an alleged history of attack by two rhinoceros sustaining injuries over the abdomen, chest, shoulder and thigh.

**Clinical Findings and Investigations:**

There was a lacerated abdomen with evisceration of the stomach, small intestine, transverse colon and omentum and a lacerated wound over the left lateral thigh and left buttock along with the right shoulder. Extended Focused Assessment with Sonography in Trauma ultrasound showed minimal free fluid in the pelvis. Blood profile revealed reduced haemoglobin and deranged prothrombin time/international normalized ratio.

**Interventions and Outcome:**

Patient underwent exploratory laparotomy twice with repair of diaphragmatic injury, excision of avulsed greater omentum in the first setting and repair of gastric perforation in the second setting with stable haemodynamic status.

**Conclusion:**

Abdominal evisceration injury by rhinoceros attack is life threatening though rare. Its management should consist of assessing for and controlling associated haemorrhage, assessing for bowel content leakage, covering the eviscerated abdominal contents and early reduction of viscera if there is no active bleeding.

HIGHLIGHTS

- Injuries and death from animal attacks have become global issues at present.
- Rhinoceros assault on humans causes minor to major extensive life-threating injuries.
- Early prehospital care and meticulous intervention by the multidisciplinary team is a must for the survival of patients following animal attacks on humans.
- Exploratory laparotomy with damage control surgery and reduction of viscera following haemostasis takes the primary space in the management of evisceration.

## Introduction

Injuries and death from animal attacks continue to be one of major public health problems worldwide at present. Although the majority of such injuries are minor, however, many injuries remain undocumented and many people die, especially in third-world countries like Nepal, India^1^. Animal-related attacks and injuries should be analysed based on the behaviour and anatomy of the attacking animal. Rhinoceros-related injuries in humans are very rare till date^2^.

Attacks on people by wild animals in and around protected conservation areas, where the same resources are shared by people and wildlife, are one of the major conservation challenges in getting effective public support. The majority of wildlife attacks occur in forests and cultivated land, particularly in a country like Nepal, where they share the same land with animals and local residents. Also, most people are unaware of the behaviour of animals that attacked. Mostly the concentration of the attacks occurred with less than one victim per square kilometre^3^.

Herein we report a rare case of a 36-year-old male, serving soldier, with a lethal open lacerated abdomen with evisceration of the stomach, transverse colon, omentum and small intestine with diaphragmatic laceration along with a lacerated wound over the left buttock region, left lateral thigh and right shoulder following an attack by a wild rhinoceros. This report has been reported in line with the Surgical CAse REport (SCARE) 2020 criteria^4^.

## Method

We reported this case following the updated consensus-based Surgical CAse REport (SCARE) Guidelines^4^.

## Case presentation

**Figure 1:**
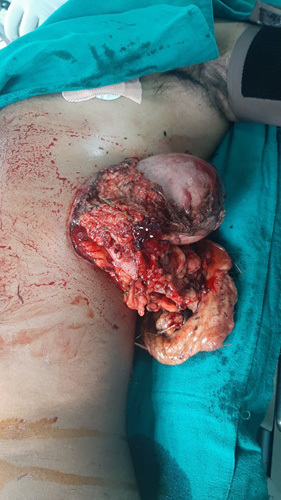
Lacerted wound over the left lateral hypochondriac region with evisceration of the stomach, small intestine omentum and transverse colon.

A 36-year-old male, regular serving soldier, with no known comorbidities, alleged history of attack by two rhinos while on a regular patrol in the Chitwan forest at around 1 p.m. on 24 September 2022 was heli-rescued to the emergency department of our centre with sustained injuries over chest, abdomen and thigh. There was a lacerated abdomen with exposed viscera with active bleeding along with laceration over the left buttock, left lateral thigh and right shoulder (Fig. 1). There was no history of loss of consciousness, bleeding from the eye, nose and throat, also no vomiting, fever and abnormal body movement. On clinical examination, the patient’s general condition is grossly fair and is oriented to time, place and person, with Glasgow Coma Score being 15/15. His past medical, surgical and allergic history is unremarkable. He is a nonsmoker and does not consume alcohol.

On examination of his vital parameters, he had an elevated pulse rate of 130–150 bpm and an elevated respiratory rate of 25 breaths/minute with a blood pressure of 110/60 mmHg and was kept in facemask due to decrease in SpO_2_. Local examination shows an open lacerated abdominal wound over the left lateral hypochondriac and lumbar region with evisceration of viscera containing the stomach, transverse colon, omentum and small intestine. Approximately a 8×5 cm lacerated wound is present over the left buttock region, a 6×5 cm lacerated wound over the left lateral thigh region and a 4×4 cm lacerated wound present over the right shoulder region.

The laboratory analysis showed a mild decrease in haemoglobin 10.8 g/dl and a slight increase in total leucocyte count 12,100 cells/cumm with prothrombin time/international normalized ratio value 18.4/1.4 and increase in amylase 107 U/l, alkaline phosphatase 97 U/l and serology was nonreactive for HIV, hepatitis B and C. Extended Focused Assessment with Sonography in Trauma ultrasound shows minimal free fluid in the pelvis, no fluid in pleural and pericardial space, hepatorenal pouch, left and right para colic gutters with limited scan.

**Figure 2:**
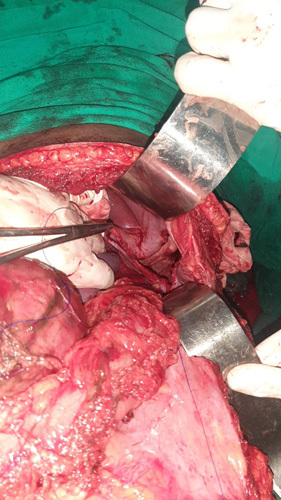
Intraoperative findings during exploratory laparotomy.

Primary management was done in the emergency room with resuscitating intravenous (i.v.) fluids ringer lactate, injection i.v. antibiotics piperacillin and tazobactam and tranexamic acid. And after counselling and consent, the patient was shifted to the operation theatre for explorative laparotomy, and rapid sequence intubation was done due to the risk of aspiration; central venous pressure line was inserted into the right internal jugular vein under ultrasonography guidance, injection fentanyl and noradrenaline were given to maintain MAP more than 65 mmHg and 1 whole pint blood and 1 pint packed RBC with 10 mg furosemide in between was given intraoperatively. The patient underwent exploratory laparotomy with repair of diaphragmatic injury, closure of chest wall over chest tube, excision of avulsed greater omentum, debridement of chest wall wound with retaining suture and peritoneal lavage was done with two drains in situ (Fig. 2). The patient was shifted to the surgical ICU with endotracheal tube, abdominal drain and chest drain and kept in a mechanical ventilator under inotropic support and daily debridement of the wound was done under IVA. The patient was extubated on first postoperative day and again re-intubated on 3rd postoperative day following which he developed pneumonia, acute respiratory distress syndrome and arterial blood gas report showed severe metabolic acidosis and the patient was referred to another centre for further critical care and management where he was given ICU care and underwent re-explorative laparotomy for gastric perforation and repair of gastric perforation with peritoneal lavage was done. The patient was extubated on the 3rd postoperative day, and the chest tube was removed on the 9th of October 2022. On the patient party’s request, the patient was again shifted to our centre and was haemodynamically stable. Chest physiotherapy and incentive spirometry continued along with ICU care with a multidisciplinary team. He developed a surgical site infection, and i.v. antibiotics were given as per culture and sensitivity report along with twice daily dressing. Secondary suturing of the right subcostal wound was done on 17th October 2022. All of his abdominal drains were removed as per daily monitoring of drain secretion. With further closure observation and management, he was asymptomatic and haemodynamically stable, and he regained his health to an optimal level and was discharged. On his scheduled follow-up, he was free from any symptoms.

## Discussion

Animal-related injuries like bite, claw wounds, goring from a pet or wild animals are most common causes of morbidity and mortality particularly in the rural tropics, third-world countries and discussed as neglected public health problems throughout the world. Animal-related injuries are known to be more common in rural areas^1^. Most of the studies conducted about wildlife attacks on the human population so far have disproportionately focused on serious fatal attacks, but further exploration and study is needed to understand other injury severities of injuries (minor, serious, death). In the context of Chitwan National Park, people suffer from attacks by various wild animals such as rhino (*Rhinoceros unicornis*), tiger (*Panthera tigris*), sloth bear (*Melursus ursinus*), elephant (*Elephas maximus*) and wild boar (*Sus scrofa*)^5^.

Wild animal attacks are responsible for an array of potentially lethal injuries, including both blunt force injuries and penetrating injuries characteristically involving larger animals such as rhinoceros, elephants, and cattle horses that may kick, crush, goring or trample a victim, causing head, chest and abdominal injuries.

Injuries caused by this type of attack by animals, both wild and domestic can be associated with extensive soft-tissue damage despite externally appearing to be simple puncture wounds and may have had developed severe deeper wounds inside body cavity and viscera^6^.

Farm workers and security personnel are, in particular, at high risk of lethal injuries in third-world countries like Nepal. Injury and death from wildlife attacks often result in people feeling violent resentment and hostility against the wildlife involved and, therefore, may undermine public support for conservation. Although a country like Nepal, rich in biodiversity, is doing well in its conservation efforts, human–wildlife conflicts have been a major challenge in recent years. Patterns were examined of human injury and death caused by large mammals using data from attack events and their spatiotemporal dimensions collected from a national survey of data available in Nepal. In the tropics of Chitwan National Park, one-horned rhinoceros and bears had a higher frequency of attacks than Bengal tigers; tigers caused more fatalities than each of these two species^7^. The human–wildlife conflicts causing nuisances and grievous injuries are becoming a growing public health concern over recent years worldwide^7^. The attacks on humans by big captive felids have been an issue of concern for the administration of zoological parks and wildlife conservationists. The theme of human–animal conflict takes a new dimension for the wild animals kept in zoos, circuses, exotic animal farms, and private custody. Despite the potential dangers involved, the zookeepers, security personnel and guards have to closely interact with the captive animals for catering to the needs of food, general health and well-being. The literature has described cases of attacks by captive animals resulting in the death of the primary caretaker^8^.

From previous case reports, it was shown that very few studies have been done worldwide regarding injuries caused by the attack of rhinoceros to humans, thus showing the need for a study and literature review regarding the pattern of injuries caused by animal attacks and solving issues of human–animal conflicts. Rhinoceroses have no natural predators. Male rhinoceroses protect their exclusive nonoverlapping territories that they mark by the smell of their own urine and stool. Dominant male rhinoceroses wipe their horns to trees or the ground and then attack each other from a distance by touching their horns while their heads and ears are raised up. The reaction of rhinoceros to humans varies. It depends mainly on sensory responses. The animal usually prefers not to attack humans but to run for a few hundred metres before stopping. The rhinoceros-related human injury, which was reported by Shah *et al*.^1^ was mainly a goring penetrating chest injury that caused a large left open wound.

Our patient was also presented with grievous life-threatening lacerated injury after an attack by two rhinoceros in Chitwan National Park. Although it was previously reported that the chest was the most commonly injured human region following a rhino attack which is attributed that to the height of the rhinoceros and the position of its horn, but our patient presented with the gross clinical presentation of a lacerated wound over left lateral thigh and buttock region with active bleeding and open lacerated abdominal wound over left lateral hypochondriac and lumber region with evisceration of hernia containing the stomach, transverse colon, small intestine and omentum. And after meticulous reading of the computed tomography scan and correlating with the associated history and clinical presentation, a re-explorative laparotomy directed us to the diagnosis of gastric perforation in our patient. Though the vital status of the patient was deranged, his general appearance was grossly fair, which is usually not a common presentation. As described in the above paragraph about the lethal status of the patient, it showed that after explorative laparotomy and re-explorative laparotomy, meticulous care and treatment from a multidisciplinary team promptly could save a life from the door of death.

## Conclusion

Although very few rhinoceros attack-related injuries in humans are documented and reported, it should be concerning that life-threatening traumatic injuries occur after animal assault. General public awareness is mandatory regarding different kinds of injuries and the nature of animals residing in the vicinity. If in any way humans are attacked by animals, the best way to save human life is through immediate and prompt management at the site of injury or, if possible, taking the victim to nearby health centres or hospitals. As in our case, though the injuries were life threatening and grievous, prompt and meticulous management by healthcare professionals at the tertiary level enabled hospitals to prevent loss of life and give the patient an optimal level of health.

Initial management and prognosis of patients with abdominal evisceration start from initial prehospital care providers to approaches made by surgeons and efforts from the multidisciplinary team. Thus it should consist of assessing for and controlling associated haemorrhage, assessing for bowel content leakage, covering the eviscerated abdominal contents with a moist, sterile barrier, and carefully reassessing the patient. Mortality in abdominal evisceration is more likely to be secondary to associated injuries than to the evisceration itself. Even after a primary intraoperative survey, one may miss other grievous injuries when it comes to animal attacks and require a second surgery. Thus during the immediate initial management of such a case, even though a minor injury is visible externally, one must suspect lethal internal injuries.

## Ethical approval

Not applicable.

## Patient consent

Written informed consent was obtained from the patient for the publication of this case report and accompanying images. A copy is available for review by the Editor in chief of this journal on request.

## Sources of funding

No funding was required for the publication of this case report.

## Author contribution

S.K. and M.S.: led data collection, contributed to writing the case information and discussion, and contributed to the process of original draft preparation. S. Basukala, S.M., S. Banmala, A.H., N.T., and B.B.T. contributed to conceptualization, methodology, and discussion and contributed to the process of original draft preparation. All the authors approved of the final version of the manuscript and agreed to be accountable for all aspects of the work, ensuring questions related to the accuracy or integrity of any part of the work are appropriately investigated and resolved.

## Conflicts of interest disclosure

None of the authors have any conflicts of interest to disclose.

## Guarantor

S. Karki.

## Provenance and peer review

Not commissioned, externally peer reviewed.

## Data availability statement

Not applicable.
